# Necrotizing Pancreatitis Associated With an Ingested Screw Adjacent to the Ampulla of Vater

**DOI:** 10.14309/crj.0000000000002340

**Published:** 2026-09-28

**Authors:** Francis Sangwon Lee, Christina Lee, Clare Lee, Vincent Huntley

**Affiliations:** 1 Department of General Surgery, Sutter Coast Hospital, Crescent City, CA; 2 Department of Communication Sciences and Disorders, Columbia University, New York, NY; 3 Department of Anthropology, Columbia University, New York, NY

**Keywords:** necrotizing pancreatitis, foreign body ingestion, metallic screw, periampullary foreign body, endoscopy

## Abstract

A 78-year-old man presented with severe epigastric pain and was diagnosed with acute pancreatitis that progressed to necrotizing pancreatitis despite supportive management. No common etiologies were identified. During endoscopic nasojejunal feeding tube placement, a metallic screw was incidentally discovered in the second portion of the duodenum adjacent to the major papilla and successfully removed. The foreign body had not been detected on previous computed tomography, ultrasound, or abdominal radiography. The patient recovered completely without recurrence. This case highlights a rare periampullary foreign body in otherwise unexplained pancreatitis and raises the possibility of a mechanical contribution to disease pathogenesis.

## INTRODUCTION

Acute pancreatitis is a common gastrointestinal disorder with gallstone disease and alcohol use representing the most frequent etiologies.^1^ Less common causes include hypertriglyceridemia, medications, hypercalcemia, autoimmune disease, trauma, malignancy, and structural pancreaticobiliary abnormalities.^1^ Necrotizing pancreatitis represents a severe form of acute pancreatitis associated with substantial morbidity and mortality.

Foreign body ingestion is a rare and underrecognized cause of pancreatitis. Most ingested objects pass through the gastrointestinal tract uneventfully. In uncommon circumstances, however, foreign bodies may contribute to pancreatic inflammation through direct penetration of the gastrointestinal tract into adjacent pancreatic tissue or through mechanical irritation and obstruction involving the major papilla or sphincter of Oddi.

Most previously reported cases involve sharp objects such as fish bones, sewing needles, toothpicks, and dental prostheses causing penetrating injury into the stomach, duodenum, or pancreas itself.^2–4^ Less commonly, foreign bodies have been reported to obstruct or irritate the ampullary region, impairing pancreatic drainage and precipitating pancreatitis.^2,4^ By contrast, the present case involved a blunt metallic screw located adjacent to the major papilla without evidence of direct pancreatic penetration, suggesting a potentially different mechanism involving intermittent periampullary obstruction, duodenal spasms, or localized pancreatic sphincter irritation.

To our knowledge, a review of the literature over the past 13 years identified no reported cases of severe necrotizing pancreatitis associated with an ingested metallic screw. This case may therefore represent a previously unreported presentation of foreign body-associated pancreatitis and suggests a potential mechanism of pancreatic injury distinct from the penetrating injuries more commonly described.

## CASE REPORT

A previously healthy 78-year-old man presented to the emergency department on day 1 with severe epigastric abdominal pain radiating to the back associated with nausea and poor oral intake. Initial vital signs demonstrated blood pressure 181/79 mm Hg, heart rate 79 beats/min, respiratory rate 16 breaths/min, oxygen saturation 94% on room air, and temperature 36.6°C.

Medical history was significant only for prostate cancer treated successfully with chemotherapy approximately 4 years earlier. The patient denied alcohol use, previous pancreatitis, gallbladder disease, pancreatic disease, diabetes mellitus, peptic ulcer disease, gastroesophageal reflux disease, chronic opioid use, hypercalcemia, or other malignancy. He was taking no medications other than daily vitamins and was a nonsmoker.

Initial laboratory evaluation demonstrated lipase 3,905 U/L with normal liver function studies including aspartate aminotransferase 36 U/L, alanine aminotransferase 47 U/L, alkaline phosphatase 65 U/L, and total bilirubin 0.5 mg/dL. Triglycerides were mildly elevated at 224 mg/dL, although the patient had experienced poor oral intake for several days before presentation. Serum albumin was decreased at 2.0 g/dL. Initial white blood cell count was 10 ×10^9^/L and lactate was 2.2 mmol/L.

Abdominal and pelvic computed tomography (CT) imaging obtained on day 1 demonstrated pancreatic edema with extensive peripancreatic inflammatory change extending into the left paracolic gutter consistent with acute pancreatitis. No pancreatic ductal dilation, gallstones, biliary dilation, or foreign body were identified. Right upper quadrant ultrasound performed the same day demonstrated no gallstones, no gallbladder wall thickening, and a common bile duct measuring 6 mm.

The patient was admitted to the intensive care unit and managed with aggressive intravenous fluid resuscitation, bowel rest, intravenous analgesia, and supportive care. On day 2, the patient developed worsening leukocytosis and low-grade fever to 37.9°C. White blood cell count subsequently increased to 17.4 ×10^9^/L by day 2, while lactate increased to 3.1 mmol/L before improving to 1.9 mmol/L following initiation of empiric piperacillin-tazobactam therapy later in day 2. Although antibiotics are not typically indicated in sterile necrotizing pancreatitis, the managing hospitalist initiated empiric therapy given clinical concern for possible infected necrosis.^1^

Serial lipase measurements demonstrated progressive improvement with values decreasing from 3,905 U/L on admission day 1 to 1,272 U/L on day 2 and further decreasing to 91 U/L by day 5. Despite improving laboratory values, the patient continued to experience persistent abdominal pain and inability to tolerate oral intake.

Repeat CT imaging performed on day 5 demonstrated voluminous inflammatory pancreatic changes with findings consistent with evolving necrotizing pancreatitis. The gallbladder remained unremarkable, and the common bile duct again measured 6 mm without evidence of obstruction. Mild dilation of the upper small bowel was also noted. No foreign body was visualized on repeat imaging.

Due to persistent inability to tolerate oral intake after 5 days, upper endoscopy with enteric feeding tube placement was performed on day 6. During endoscopy, a metallic machine-thread screw measuring approximately 1 cm in length and 3 mm in width was incidentally identified within the C-loop of the duodenum between the second and third portions adjacent to the major papilla. The screw did not appear to directly obstruct the ampullary orifice and no significant periampullary inflammatory changes were documented. The foreign body was successfully removed using biopsy forceps without complication.

Notably, the foreign body had not been visualized on 2 CT scans, abdominal radiography, or abdominal ultrasound performed during the hospitalization. One possible explanation is the small width of the screw, measuring approximately 3 mm, while CT cross-sectional slice thickness was reportedly up to 5 mm. Depending on orientation within the duodenum, the screw may have been obscured between imaging slices. Surrounding inflammatory changes, positioning within duodenal folds, and adjacent bowel contents may have further limited detection.

Following endoscopic removal, the patient experienced progressive improvement in abdominal pain and clinical status. Upon being shown the retrieved object, the patient recalled frequently holding screws between his teeth while working as a mechanic, although he did not specifically remember swallowing the object.

Although a direct causal relationship cannot be definitively established, no alternative etiology for pancreatitis was identified. The periampullary foreign body remained the only identifiable abnormality potentially capable of contributing to pancreatic inflammation.

## DISCUSSION

Foreign body-associated pancreatitis is exceedingly rare. Proposed mechanisms generally fall into 2 categories: direct penetration of the gastrointestinal tract into adjacent pancreatic tissue by sharp objects, and mechanical obstruction or irritation involving the major papilla or sphincter of Oddi.^2–5^

Most previously reported cases involve sharp foreign bodies such as fish bones, sewing needles, toothpicks, and dental prostheses causing penetrating injury into the stomach, duodenum, or pancreatic tissue.^2,3,5^ Less commonly, foreign bodies have been reported to obstruct or irritate the ampullary region, impairing pancreatic drainage and precipitating pancreatitis.^2,4^ A notable report described migration of an orthodontic Kobayashi ligature into the major papilla resulting in chronic pancreatitis, further supporting the potential role of ampullary obstruction in pancreatic inflammation.^4^

In our review of the literature, we found no previous reports of severe necrotizing pancreatitis associated with an ingested metallic screw. The present case differs from previously reported cases in that the foreign body was blunt and located adjacent to the major papilla, without evidence of direct pancreatic penetration or confirmed ampullary impaction.

In the present case, the metallic screw was not visualized within the ampullary orifice itself, limiting the ability to definitively establish causality. However, several findings raise suspicion for a possible association. The patient lacked common pancreatitis risk factors including alcohol use, smoking, gallstone disease, pancreaticobiliary pathology, hypercalcemia, medication-associated pancreatitis, or significant hypertriglyceridemia. Liver function tests remained within normal limits throughout hospitalization, and both ultrasound and CT imaging repeatedly demonstrated an unremarkable gallbladder and normal-caliber common bile duct.

The screw was located immediately adjacent to the major papilla within the second portion of the duodenum. One possible mechanism is intermittent transient obstruction or spasm of the pancreatic sphincter segment caused by movement of the foreign body within the periampullary duodenum. Localized irritation or intermittent impairment of pancreatic drainage due to spasms or obstruction can contribute to the development of pancreatitis despite the absence of direct ampullary impaction at the time of endoscopy.^6,7^ Notably, the patient demonstrated pancreatic inflammation without laboratory evidence of biliary obstruction, as liver function studies and bilirubin levels remained within normal limits throughout hospitalization. This finding raises the possibility that the foreign body may have selectively affected the pancreatic ductal component of the sphincter of Oddi while sparing the biliary outflow tract.^6,7^

An additional notable feature of this case was the failure of 2 CT scans, abdominal radiography, and ultrasound imaging to identify the metallic foreign body before endoscopy. Metallic objects are typically radiopaque and detectable on imaging; however, several factors may explain the missed detection in this case. The screw measured only approximately 3 mm in width, while CT cross-sectional slice thickness can be up to 5 mm, potentially allowing the object to be obscured between imaging slices depending on its orientation within the duodenum. In addition, surrounding inflammatory changes, positioning within duodenal folds, and adjacent bowel contents may have further limited visualization. Retrospectively, dedicated plain abdominal radiography with focused review for small metallic foreign bodies may have improved detection.

Importantly, this case does not prove that the metallic screw caused necrotizing pancreatitis. The patient also received supportive management including bowel rest, intravenous fluids, analgesia, antibiotics, and nutritional support, all of which may have contributed to eventual clinical improvement. Nevertheless, in the absence of another identifiable etiology, the temporal association between foreign body removal and subsequent clinical recovery raises the possibility that the periampullary screw may have been a contributing factor (Figure 1).

**Figure 1.**
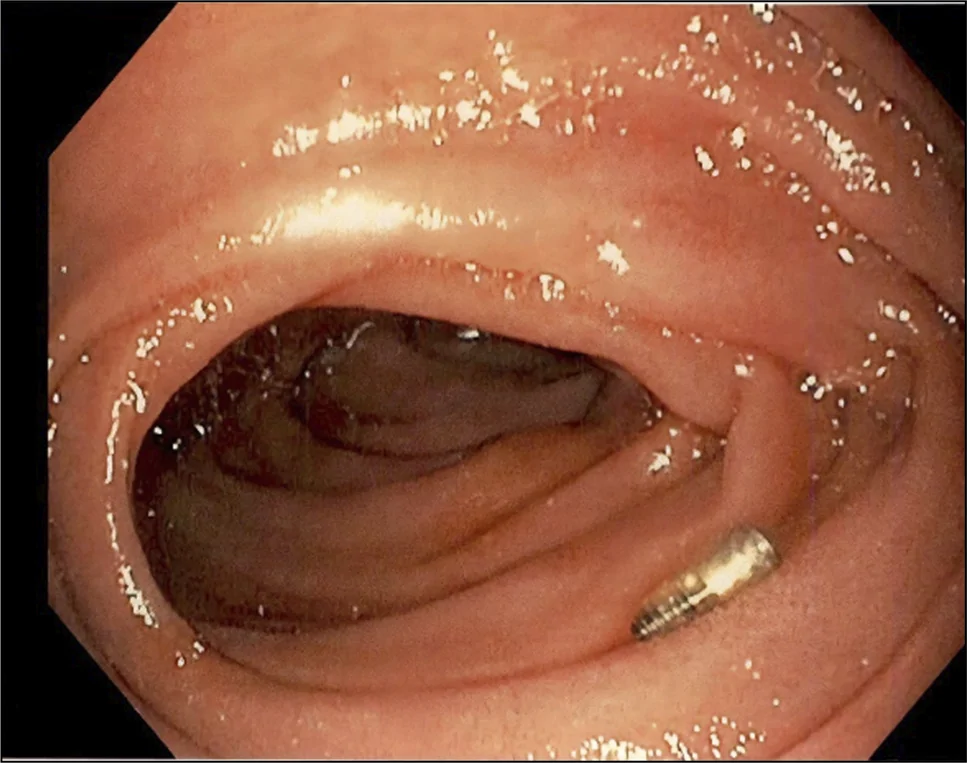
Metallic screw visualized within the second portion of the duodenum adjacent to the major papilla during upper endoscopy before removal.

This case highlights a rare and unusual potential etiology of necrotizing pancreatitis involving an ingested metallic screw located adjacent to the ampulla of Vater. Although definitive causality cannot be established, the absence of common pancreatitis risk factors, the periampullary location of the foreign body, and the patient's subsequent recovery following endoscopic removal support the possibility that intermittent mechanical irritation or transient pancreatic outflow obstruction contributed to the inflammatory process. This report also emphasizes the diagnostic limitations of standard imaging modalities in detecting small metallic foreign bodies within the gastrointestinal tract. Clinicians should maintain a broad differential diagnosis and remain mindful of occult foreign body ingestion in patients with otherwise unexplained pancreatitis, particularly when conventional etiologies have been excluded.

## DISCLOSURES

Author contributions: FS Lee: Conception and design of the study, clinical management of the patient, endoscopic procedure, data collection, drafting and critical revision of the manuscript. C. Lee: Literature review, manuscript drafting, and critical revision of the manuscript. Cl. Lee: Data collection, literature review, and manuscript revision. V. Huntley: Clinical management of the patient, data collection, and manuscript revision. F.S. Lee is the article guarantor.

Financial disclosure: None to report.

Informed consent was obtained for this case report.

## ABBREVIATIONS:

CT: computed tomography
EGD: esophagogastroduodenoscopy
CBD: common bile duct
ICU: intensive care unit
NFMFB: non-ferromagnetic metal foreign body
